# Supplementary material for: Overt visual attention modulates decision-related signals in the frontal cortex
Source: eLife. 2026 Aug 19;14:RP103846. doi: 10.7554/eLife.103846 (PMC13489626; doi:10.7554/eLife.103846)
Supplement: Peer review history

# Editor report: eLife Assessment

**DOI:** 10.7554/eLife.103846.4.sa0

**Authors:** Redmond G O'Connell

**Affiliations:** Trinity College Dublin Ireland

**Keywords:** Convincing, Important

This **important** work examines the effects of gaze on valuation signals in the human brain as participants choose between bundles of sequentially presented items food items. The paper provides **convincing** analyses of how gaze affects participants choice behaviour and how this varies across time. The work will be of interest to neuroscientists working on attention and decision-making.

# Referee report: Reviewer #1 (Public review)

**DOI:** 10.7554/eLife.103846.4.sa1

[Editors' note: this version has been assessed by the Reviewing Editor without further input from the original reviewers. The authors have updated the labelling of Figure 7. As all of the reviewer comments have now been addressed, I believe that this version of the manuscript can now be put forward as the Version of Record.]

Summary:

This study builds upon a major theoretical account of value-based choice, the 'attentional drift diffusion model' (aDDM), and examines whether and how this might be implemented in the human brain using functional magnetic resonance imaging (fMRI). The aDDM states that the process of internal evidence accumulation across time should be weighted by the decision maker's gaze, with more weight being assigned to the currently fixated item. The present study aims to test whether there are (a) regions of the brain where signals related to the currently presented value are affected by the participant's gaze; (b) regions of the brain where previously accumulated information is weighted by gaze.

To examine this, the authors developed a novel paradigm that allowed them to dissociate currently and previously presented evidence, at a timescale amenable to measuring neural responses with fMRI. They asked participants to choose between bundles or 'lotteries' of food times, which they revealed sequentially and slowly to the participant across time. This allowed modelling of the haemodynamic response to each new observation in the lottery, separately for previously accumulated and currently presented evidence.

Using this approach, they find that regions of the brain supporting valuation (vmPFC and ventral striatum) have responses reflecting gaze-weighted valuation of the currently presented item, where as regions previously associated with evidence accumulation (preSMA and IPS) have responses reflected gaze-weighted modulation of previously accumulated evidence.

A major strength of the current paper is the design of the task, nicely allowing the researchers to examine evidence accumulation across time despite using a technique with poor temporal resolution. The dissociation between currently presented and previously accumulated evidence in different brain regions in GLM1 (before gaze-weighting), as presented in Figure 5, is already compelling. The result that regions such as preSMA response positively to |AV| (absolute difference in accumulated value) is particularly interesting, as it would seem that the 'decision conflict' account of this region's activity might predict the exact opposite result. Additionally, the behaviour has been well modelled at the end of the paper when examining temporal weighting functions across the multiple samples.

In response to reviewer comments, the authors have explicitly tested for the effects of gaze-weighting over and above any main effect of value, and convincingly shown that these effects are both present in the main regions of interest - namely |SV| and gaze-weighted |SV| in the vmPFC, alongside |AV| and |AV_gaze| in the pre-SMA. This provides clear evidence in support of the notion of gaze-weighting of value signals in these regions.

# Referee report: Reviewer #2 (Public review)

**DOI:** 10.7554/eLife.103846.4.sa2

Summary:

In this paper the authors seek to disentangle brain areas that encode the subjective value of individual stimuli/items (input regions) from those that accumulate those values into decision variables (integrators) for value-based choice. The authors used a novel task in which stimulus presentation was slowed down to ensure that such a dissociation was possible using fMRI despite its relatively low temporal resolution. In addition, the authors leveraged the fact that gaze increases item value, providing a means of distinguishing brain regions that encode decision variables from those that encode other quantities such as conflict or time-on-task. The authors adopt a region-of-interest approach based on an extensive previous literature and found that the ventral striatum and vmPFC correlated with the item values and not their accumulation whereas the pre-SMA, IPS and dlPFC correlated more strongly with their accumulation. Further analysis revealed that the pre-SMA was the only one of the three integrator regions to also exhibit gaze modulation.

The study uses a highly innovative design and addresses an important and timely topic. The manuscript is well-written and engaging, while the data analysis appears highly rigorous.

Weaknesses:

With 23 subjects the study has relatively low statistical power for fMRI although the within-subjects design and relatively high trial count reduces these concerns.

# Author comment: Author response

**DOI:** 10.7554/eLife.103846.4.sa3

**Authors:** Blair RK Shevlin, Rachael E Gwinn, Aidan Makwana, Ian Krajbich

**Affiliations:** Icahn School of Medicine at Mount Sinai New York United States; The Ohio State University Columbus United States; The Ohio State University Columbus United States; University of California, Los Angeles Los Angeles United States

The following is the authors’ response to the previous reviews

**Public Reviews:**

**Reviewer #1 (Public review):**

Summary:

This study builds upon a major theoretical account of value-based choice, the 'attentional drift diffusion model' (aDDM), and examines whether and how this might be implemented in the human brain using functional magnetic resonance imaging (fMRI). The aDDM states that the process of internal evidence accumulation across time should be weighted by the decision maker's gaze, with more weight being assigned to the currently fixated item. The present study aims to test whether there are (a) regions of the brain where signals related to the currently presented value are affected by the participant's gaze; (b) regions of the brain where previously accumulated information is weighted by gaze.

To examine this, the authors developed a novel paradigm that allowed them to dissociate currently and previously presented evidence, at a timescale amenable to measuring neural responses with fMRI. They asked participants to choose between bundles or 'lotteries' of food times, which they revealed sequentially and slowly to the participant across time. This allowed modelling of the haemodynamic response to each new observation in the lottery, separately for previously accumulated and currently presented evidence.

Using this approach, they find that regions of the brain supporting valuation (vmPFC and ventral striatum) have responses reflecting gaze-weighted valuation of the currently presented item, where as regions previously associated with evidence accumulation (preSMA and IPS) have responses reflected gaze-weighted modulation of previously accumulated evidence.

A major strength of the current paper is the design of the task, nicely allowing the researchers to examine evidence accumulation across time despite using a technique with poor temporal resolution. The dissociation between currently presented and previously accumulated evidence in different brain regions in GLM1 (before gazeweighting), as presented in Figure 5, is already compelling. The result that regions such as preSMA response positively to |AV| (absolute difference in accumulated value) is particularly interesting, as it would seem that the 'decision conflict' account of this region's activity might predict the exact opposite result. Additionally, the behaviour has been well modelled at the end of the paper when examining temporal weighting functions across the multiple samples.

In response to reviewer comments, the authors have explicitly tested for the effects of gaze-weighting over and above any main effect of value, and convincingly shown that these effects are both present in the main regions of interest - namely |SV| and gazeweighted |SV| in the vmPFC, alongside |AV| and |AV_gaze| in the pre-SMA. This provides clear evidence in support of the notion of gaze-weighting of value signals in these regions.

We thank the reviewer for their comments.

**Reviewer #2 (Public review):**

Summary:

In this paper the authors seek to disentangle brain areas that encode the subjective value of individual stimuli/items (input regions) from those that accumulate those values into decision variables (integrators) for value-based choice. The authors used a novel task in which stimulus presentation was slowed down to ensure that such a dissociation was possible using fMRI despite its relatively low temporal resolution. In addition, the authors leveraged the fact that gaze increases item value, providing a means of distinguishing brain regions that encode decision variables from those that encode other quantities such as conflict or time-on-task. The authors adopt a region-of-interest approach based on an extensive previous literature and found that the ventral striatum and vmPFC correlated with the item values and not their accumulation whereas the preSMA, IPS and dlPFC correlated more strongly with their accumulation. Further analysis revealed that the pre-SMA was the only one of the three integrator regions to also exhibit gaze modulation.

The study uses a highly innovative design and addresses an important and timely topic. The manuscript is well-written and engaging, while the data analysis appears highly rigorous.

Weaknesses:

With 23 subjects the study has relatively low statistical power for fMRI although the within-subjects design and relatively high trial count reduces these concerns.

We thank the reviewer for their comments.

**Recommendations for the authors:**

**Reviewer #1 (Recommendations for the authors):**

Something seems to have gone slightly wrong in (I think) the labelling of new figure 7 (the correlation matrix between the different regressors). There are five variables on both the x- and y-axes of the figure, and they are the same five variables - meaning the diagonal of the matrix would be all equal to 1 (being the correlation of a regressor with itself - e.g. |AVgaze| with |AVgaze|). In the figure legend, six variables are mentioned, including lagged |deltaAVgaze| - but this doesn't appear on the x or y-axes. I suspect that the authors may need to check that this matrix has been calculated correctly, and isn't being mislabelled?

We thank the reviewer for noticing this issue. We have now corrected Figure 7.
